# A soleful insight: shoe dislocation as a marker for severe injuries in car accident victims

**DOI:** 10.1186/s13054-025-05650-x

**Published:** 2025-09-25

**Authors:** Shaoyang Zhang, Jueyue Yan, Zhipeng Xu

**Affiliations:** 1 The Department of Emergency, The First Affiliated Hospital Zhejiang University School of Medicine, Qingchun Street 79th, Zhejiang Province, Hangzhou, 310003 China; 2 Department of Critical Care Medicine, The First Affiliated Hospital Zhejiang University School of Medicine, Qingchun Street 79th, Zhejiang Province, Hangzhou, 310003 China

Dear Editor,

In trauma care practice, we have observed that patients whose shoes are detached at the scene of a car accident often suffer severe injuries and have poorer prognoses. This suggests that shoe dislocation at the accident site may serve as a simple, rapid indicator for assessing injury severity. The underlying mechanism may involve force transmission through the feet and ankles during impact, causing shoes to be ejected while significant stress is applied to deeper tissues, resulting in serious internal injuries.

Typically, car accident injuries are initially assessed based on vital signs, visible wounds, and consciousness levels. However, patients with minor surface injuries may later present with hidden severe conditions such as intracranial bleeding, organ rupture, or spinal fractures (Fig. 1 A). Shoe dislodgment could act as a critical visual clue indicating a higher injury magnitude, suggesting deeper anatomical involvement, including damage to vital organs and the central nervous system.

As illustrated in Fig. 1B, we retrospectively analyzed 326 consecutive trauma patients—specifically pedestrians, cyclists, or scooter riders who were struck by motor vehicles and admitted to our trauma center between 2022 and 2024. Only patients with clearly documented shoe status in prehospital or emergency records were included to ensure data reliability. Among them, 61 patients (18.7%) had at least one shoe dislodged at the scene.Compared with those whose shoes remained intact, the shoe-dislocation group had significantly higher Injury Severity Scores (mean ± SD: 26.9 ± 9.8 vs. 14.2 ± 7.5, *p* < 0.001). They also had higher incidences of severe traumatic brain injury (GCS ≤ 8: 29.5% vs. 10.3%, *p* < 0.001), pelvic/lower extremity fractures (52.5% vs. 21.6%, *p* < 0.001), and multisystem involvement (72.1% vs. 31.8%, *p* < 0.001). These associations support the hypothesis that shoe dislocation correlates with severe trauma and can help prioritize patients for advanced care.

**Fig. 1:**
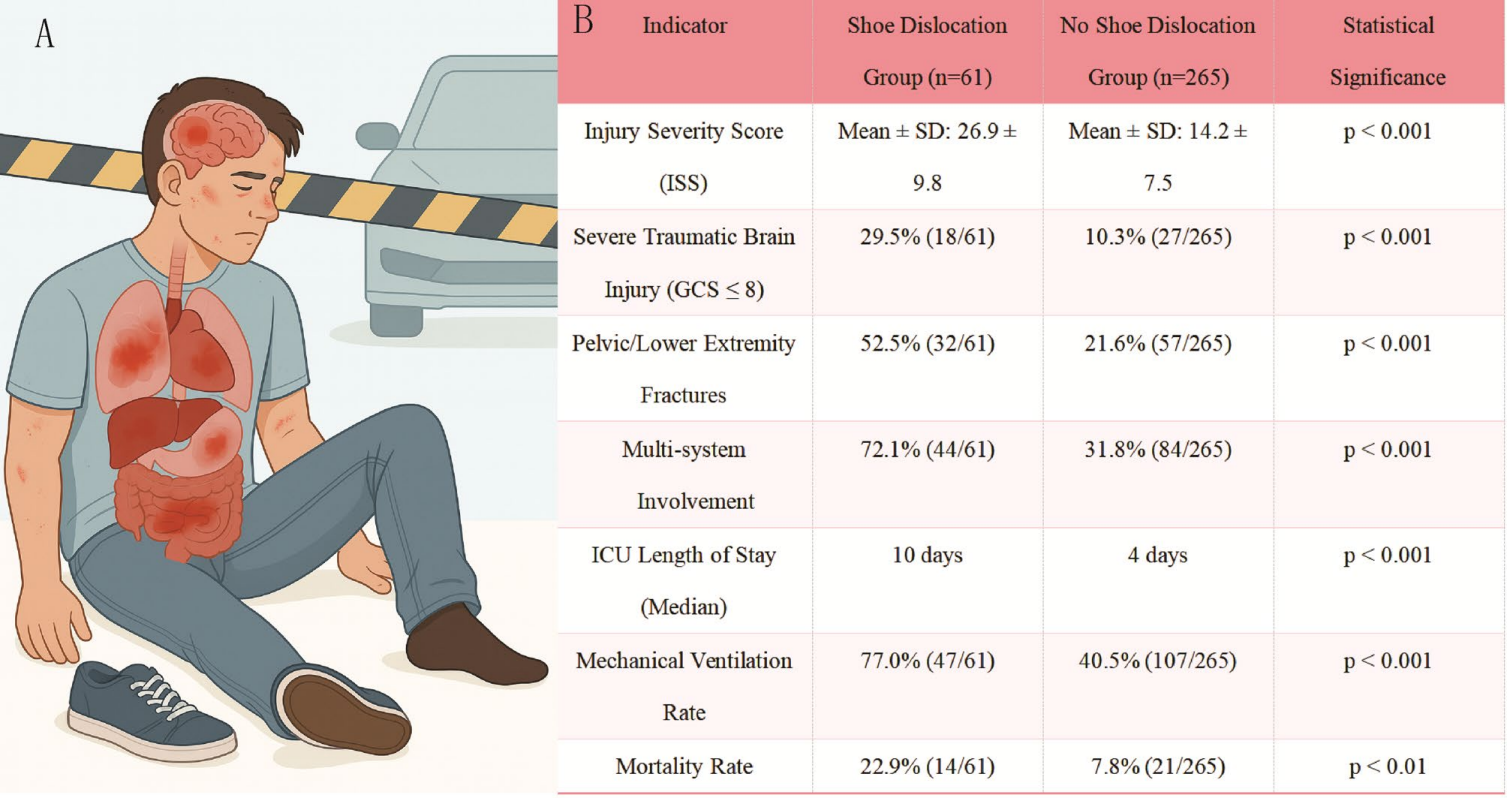
Shoe dislocation as an indicator of injury severity in motor vehicle collisions. (**A**) Trauma patient with shoe dislocation indicating severe internal injuries. (**B**) Comparison of outcomes in shoe dislocation (*n* = 61) vs. no dislocation (*n* = 265) groups post-MVC. Shoe dislocation group had higher ISS, traumatic brain injury, fractures, multi-system involvement, longer ICU stays, higher ventilation rates, and mortality (*p* < 0.001)

Prognostically, patients with shoe dislocation required longer ICU stays (median: 10 days vs. 4 days, *p* < 0.001), higher mechanical ventilation rates (77.0% vs. 40.5%, *p* < 0.001), and had a higher mortality rate (22.9% vs. 7.8%, *p* < 0.01). These findings emphasize the clinical significance of shoe dislocation as an early marker for identifying patients requiring intensive monitoring and intervention. Biomechanically, shoe dislodgment likely reflects high-magnitude forces exceeding footwear retention and energy transfer sufficient to cause multisystem tissue disruption. Thus, shoe dislocation in MVCs not only indicates potential foot and ankle trauma but may also signal early multi-organ injuries requiring urgent, specialized care.

Our findings suggest that shoe dislocation may serve as a simple, observable marker to help prioritize patients for advanced trauma care, particularly in settings requiring rapid triage. The likelihood of dislodgement can vary depending on footwear type—loose shoes like slippers or sandals may come off with minimal force. While such cases may not always indicate severe injury, they should not be dismissed, as shoe dislodgement can still reflect significant trauma. Due to the retrospective nature of our study, detailed information on shoe types was unavailable, highlighting the value of future prospective research to further refine this observation.

## Data Availability

No datasets were generated or analysed during the current study.

